# Applications of psychedelics in small doses and alternative treatments in psychiatry

**DOI:** 10.1192/j.eurpsy.2025.65

**Published:** 2025-08-26

**Authors:** K. P. Kuypers

**Affiliations:** Neuropsychology and Psychopharmacology, Maastricht University, Maastricht, Netherlands

## Abstract

**Abstract:**

Several years ago, the first studies were conducted in which the effects of psychedelics in low doses were tested in healthy participants. These studies focused mainly on the acute effects of LSD. Questions that remained unanswered were whether low doses have lasting effects, as well as whether they can reduce symptoms of ADHD. To answer these questions, we conducted two studies, administering LSD (15 mcg) repeatedly over 2 weeks to healthy participants and administering LSD (20 mcg) repeatedly over 6 weeks to participants with ADHD. The latter study was a multicenter trial conducted by Universitatsspital (Basel) and Maastricht University. Based on the findings of both studies, we have now planned a follow-up study in 100 individuals with ADHD who also have problems with emotion regulation and/or sleep, to get closer to an “average” ADHD population. This study, which will start later this year, aims to find markers of treatment response. Lastly, plans for a study investigating high-ventilation breathwork as a novel intervention for individuals with social anxiety symptoms will be presented. High-ventilation breathwork has the potential to induce altered states of consciousness similar to those elicited by psychedelics. This research aims to explore its therapeutic potential as a non-pharmacological alternative to psychedelics in psychiatric treatment.

**Disclosure of Interest:**

K. Kuypers Grant / Research support from: Part of the work that is presented is funded by Mindmed Inc (LSD, ADHD), the Beckley Foundation (LSD, healthy volunteers), and grants from the Dutch research organisation (LSD, ADHD, biomarkers) and the Tiny Blue Dot Foundation (Breathwork, Social Anxiety).

